# Translation and Validation of the Spanish Version of the Spiritual Care Competence Questionnaire (SCCQ)

**DOI:** 10.1007/s10943-021-01402-7

**Published:** 2021-08-28

**Authors:** Tania Pastrana, Eckhard Frick, Alicia Krikorian, Leticia Ascencio, Florencia Galeazzi, Arndt Büssing

**Affiliations:** 1 Department of Palliative Medicine, RWTH University Aachen, Aachen, Germany; 2 Klinik und Poliklinik für Psychosomatische Medizin und Psychotherapie, Klinikum rechts der Isar der Technischen Universität München, Munich, Germany; 3 Pain and Palliative Care Group, School of Health Sciences, Universidad Pontificia Bolivariana, Medellín, Colombia; 4 Servicio de Cuidados Paliativos, National Cancer Institute, Mexico City, México; 5 Hospice Buen Samaritano, Buenos Aires, Argentina; 6 Professorship Quality of Life, Spirituality and Coping, Institute of Integrative Medicine, Witten/Herdecke University, Gerhard-Kienle-Weg 4, 59313 Herdecke, Germany

**Keywords:** Spiritual care, Competences, Validation, Questionnaire, Translation

## Abstract

We aimed to validate the Spanish version of the *Spiritual Care Competence Questionnaire* (SCCQ) in a sample of 791 health care professionals from Spanish speaking countries coming principally from Argentina, Colombia, Mexico and Spain. Exploratory factor analysis pointed to six factors with good internal consistency (Cronbach’s alpha ranging from 0.71 to 0.90), which are in line with the factors of the primary version of the SCCQ. *Conversation competences* and *Perception of spiritual needs competences* scored highest, and *Documentation competences* and *Team spirit* the lowest, *Empowerment competences* and *Spiritual self-awareness competences* in-between. The Spanish Version of the SCCQ can be used for assessment of spiritual care competencies, planning of educational activities and for comparisons as well as monitoring/follow-up after implementation of improvement strategies.

## Introduction

Spirituality is defined as “a dynamic and intrinsic aspect of humanity through which persons seek ultimate meaning, purpose, and transcendence, and experience relationship to self, family, others, community, society, nature, and the significant or sacred” (Puchalski et al., 2014, p. 646). Spirituality—understood in such a broad sense—is recognized as part of health care, being the core aim of health care to “eliminate, reduce the impact of, or manage the varied psychological, physical, social, and spiritual experiences of illness, for both the patient and their families and communities” (Oben, 2020, p. 909). To achieve that, spiritual care must be integrated at all levels of care.

Spiritual care might be defined as a “type of care that addresses and seeks to meet existential and spiritual needs and challenges in connection with illness and crisis” (Hvidt et al., 2020, p. 2), and therefore, it is a shared responsibility of health care professionals to consider patients’ spiritual needs, resources and challenges (Frick, 2017). Spiritual care is increasingly recognized as being capable of making a positive contribution to both mental and physical health (Koenig, 2002). Additionally, spiritual care enhances patients' quality of life and coping with illness (Frick & Schießl, 2015; Gillilan et al., 2017). On the contrary, unmet spiritual concerns or needs can lead to distress and unnecessary physical and emotional suffering (Edwards et al., 2010) and have negative consequences, including poorer health status and higher cost for the system (Caldeira et al., 2013). Studies show that patients are interested in discussing spirituality in medical consultations (Best et al., 2015) and that several prefer to talk about their spiritual needs with their physicians rather than with a pastoral worker (Büssing et al., 2009), but they receive less spiritual care than desired (Fuchs et al., 2021; Kalish, 2012); additionally, a discrepancy exists in the perceptions between patients and doctors regarding what constitutes this discussion and whether it has taken place (Best et al., 2015, 2016).

Puchalski et al. (2009) advised that health care professional must be aware of the spiritual dimensions of their own lives in order to provide adequately spiritual care, and this requires processes of self-reflection. However, spiritual care is not a matter of *doing* something (even with the best intentions), but first to *listen*, what patients need and require (Büssing, 2021). Certain competences are required to meet patients’ spiritual needs. A competence is defined as “person-oriented, referring to the person's underlying characteristics and qualities that lead to an effective and/or superior performance” (McMullan et al., 2003, p. 285). Competence in spiritual care is the ability (knowledge, skills and attitudes) of the health professional to assess for and provide interventions to care for a patient’s spiritual needs (Green et al., 2020), while strengthening the resilience of health care professionals (Frick & Schießl, 2015) and improving the connection and collaboration between professional care takers and their patients (Paal et al., 2015). Spiritual care competence may correlate positively with self-efficacy (Cheng et al., 2021), while other studies did not see such associations (Frick et al., 2019). Conversely, the lack of competence is a great barrier to the provision of spiritual care (Best et al., 2016; Frick & Schießl, 2015; McSherry & Jamieson, 2011). No data are available about the number of medical/nursing schools or allied disciplines in Latin America or Spain offering courses or contents on spirituality and health in the curriculum. Lucchetti et al. (2012) found that 10% of medical schools in Brazil include this subject in the curriculum.

Spirituality and religiosity have been considered characteristic of the Latino culture in relation with health (Del Rio, 2004). A study with 221 Latin American health care professionals working in palliative care considered spirituality and religiosity very important in their lives (9/10, 0–10 and 6/10, 0–10, respectively) (Delgado Guay et al., 2018).

Several instruments have been used for the assessment of spiritual competences (Frick et al., 2019). However, such tools must be adapted to the cultural backgrounds where they are to be used. For instance, the Spirituality and Spiritual Care Rating Scale (SSCRS) had been already validated in Spanish, but its reliability was found to be low (Matthies Bornhorst, 2019). The Spiritual Care Competence Questionnaire (SCCQ) was developed to address multiprofessional self-reported spiritual care competences (Frick et al., 2019). It has been validated so far in German (Frick et al., 2019), French (Neves Oliveira, 2019) and Norwegian language (Mandelkow et al., 2021).

The aim of this study was to translate and validate the Spanish version of the SCCQ in Latin America and Spain using exploratory and confirmatory factor analysis. The validation aims to verify the applicability of this instrument to detect the competencies in spiritual care of health care professionals.

## Methods

### Study Design

An anonymous, cross-sectional study was conducted using an open online survey for health care providers in Argentina, Colombia, Mexico and Spain. A convenience, snowball sampling was used. The included Latin American countries were selected because they can be considered as representative of three subregions (Central America, Andean Region and Southern Cone) and because of their large population.

The study obtained the Institutional Review Approval (EK 144/21) and was registered as a clinical trial (CTC-A Nr. 21–141). The method and results are reported according to the checklist for Reporting Results of Internet E-Surveys (CHERRIES) (Eysenbach, 2004).

## Measures

### Spiritual Care Competence Questionnaire

The instrument uses 26 items scored with a 4-point Likert scale (0—strongly disagree, 1—disagree, 2—agree, 3—strongly agree) and differentiates 7 factors: 1) *Perception of spiritual needs* [5 items; Cronbach’s alpha = 0.82]; 2) *Team spirit* [5 items; Cronbach’s alpha = 0.81]; 3) *Documentation competences* [3 items; Cronbach’s alpha = 0.84]; 4) *Spiritual self-awareness competences* [5 items; Cronbach’s alpha = 0.83]; 5) *Knowledge about other religions* [2 items; Cronbach’s alpha = 0.73]; 6) *Conversation competences* [2 items; Cronbach’s alpha = 0.86]; 7) *Empowerment competences* [4 items; Cronbach’s alpha = 0.79].

Additional items were used as explanatory variables. Among them, four addressed barriers to spiritual care (*s44, My knowledge about religion/spirituality is too poor for me to get involved in a competent manner*; *s46, I do not have time for religious/spiritual topics*; *s47 No suitable room is available for talking privately about religious/spiritual subjects*; *s45, I do not perceive myself as an appropriate person for religious/spiritual subjects*). These cannot be regarded as competences and were combined into an additional factor (Hindrances) with acceptable internal consistency (Cronbach’s alpha = 0.72).

### Additional Variables

Apart from basic sociodemographic data (gender and age), we also included participants’ profession and area of work, years of employment, working hours per week and their professional satisfaction (5-point Likert scale: 4—very satisfied, 3—satisfied, 2—more or less satisfied, 1—not satisfied, 0—very unsatisfied).

Additional questions addressed religious orientation and whether participants regard themselves as a believing person (3—yes, indeed, 2—yes, somehow, 1—rather not, 0—not at all), and if and how often they meditated or prayed (3—yes, on a regular base; 2—occasionally, 1—rather rarely, 0—not at all).

### Translation

The translation process was modified from that proposed by (Beaton et al., 2000) and follows the recommendations of Koenig and Al Zaben (2021). It consisted of a translation from the original SCCQ in German into Spanish (forward translation) and a back-translation into German by native speakers. Psychometric properties were tested with 157 participants (Matthies Bornhorst, 2019). A group of bilingual researchers (EF and TP and an expert in modern languages, including one of the developer of the SCCQ), reviewed the translations and inconsistences with the original meaning developing a new draft (harmonization). Then cognitive debriefing were conducted with experts in spirituality in each of the Spanish language target countries, attending that the language used had the similar meaning in each country. The final draft was reviewed again with the bilingual researchers and the other author of the questionnaire (TP, EF and AB) (review of cognitive debriefing). Finally, all researchers reviewed the final draft (proofreading) and few remaining changes were performed.

The survey was uploaded in LimeSurvey® and distributed in three pages (introduction/informed consent, sociodemographic questions and SCCQ). Its technical functionality was tested before its administration. Participants were provided with general information about the study as well as the objectives of the survey and length of time of the survey (less than 15 min). The informed consent was located at the starting page of the online survey with additional information about storage, anonymity and confidentiality. The survey was completely voluntary with no incentives offered, nor use of advertising.

The invitation and link for the questionnaire was distributed broadly through contacts with potential participants from health care (individuals, networks, emails and scientific societies), and it was asked to forward it to other potential participants (snowball). The survey was opened on May 28 and data collection was closed on June 30, 2021.

Responses were captured automatically through the survey web app. No consistency or completeness checks before the submission was done, neither was it possible to review and change answers after advancing from one page to another.

### Statistical Methods

Descriptive statistics, internal consistency (Cronbach’s coefficient *α*) and factor analyses (principal component analysis using Oblimin rotation with Kaiser’s normalization) as well as first-order correlations (Spearman’s *ρ*) were computed with IBM SPSS Statistics for Windows, Version 23.0. Given the exploratory character of this study, the significance level was set at *p* < 0.01. Spearman correlation *r* > 0.5 was considered strong, *r* between 0.3 and 0.5 as a moderate correlation, r between 0.2 and 0.3 as a weak correlation and *r* < 0.2 as negligible or no correlation.

Religious orientation was dichotomized in believers (3—yes, indeed, 2— yes, somehow) and nonbelievers (1—rather not, 0—not at all).

To evaluate the validity of the theoretical factor model defined firstly for the German sample for this dataset, a confirmatory factor analysis (CFA) was performed. This multivariate statistical method is applied as a confirmation step to validate and in some cases replicate the findings from theory or other related studies. To evaluate the appropriateness of the dataset to a factor analysis, the Bartlett’s test and the Kaiser–Meyer–Olkin (KMO) statistics were used. The sample is adequate for the factor analysis if the p-value from Bartlett’s test is smaller than 0.05 and if KMO is equal or superior to 0.6. We worked with R 4.0.2 and package lavaan (Rosseel, 2012) to perform the confirmatory factor analysis. We evaluated the quality of the model based on those fit statistics: root mean square error of approximation (RMSEA), standardized root mean square residual (SRMR), comparative fit index (CFI) and Tucker–Lewis index (TLI). The thresholds for a good fit are CFI and TLI > 0.95, SRMR < 0.06 and RMSEA < 0.05.

## Results

### Sociodemographic Details of Participants

A total of 1001 persons (75% women) with a mean age of 45.3 ± 12.3 years visited the survey and answered the first survey page, and 791 completed the SCCQ (Completion rate = 79%). Only completed questionnaires were included in the analysis. Both “Starters” and “Completers” did not significantly differ with respect to gender, age, religious denomination, years in their job or recruiting country.

Responders were 73% women, aged 45.4 ± 11.9 and came from various countries, predominantly from Mexico (38.9%) followed by Spain (12.6%), Argentina (11.6%) and Colombia (10.6%). Most of the completing participants were physicians (45%), followed by other therapists (13%) and nurses (13%). They were working predominantly in the field of palliative care (37%) and general medicine (16%) with an average job satisfaction of 82.0 ± 19.4 (Table 1).

**Table 1:** Sociodemographic data of participants (*n* = 792)

Variable	%/mean
*Gender (%)*	
Women	72.8
Men	27.2
*Mean age (years)*	45.4 ± 11.9
*Work experience (years)*	18.0 ± 11.4
*Weekly working time (hours)*	42.4 ± 18.1
Profession (%)*	
Physician	45.0
Therapist	13.1
Nurse	12.9
Psychologist	2.2
Chaplain	0.9
Other	10.0
Departments (%)*	
Palliative care	37.2
General medicine	16.0
Pediatrics	9.3
Internal medicine	5.6
Geriatrics	4.4
Psychiatry/psychotherapy	2.9
Surgery/orthopedic	1.8
Gynecology/obstetrics	1.3
Other	39.0
*Job satisfaction [0–100]*	82.0 ± 19.4
*Countries (%)*	
Mexico	38.9
Spain	12.6
Argentina	11.6
Colombia	10.6
Ecuador	10.5
Chile	4.2
Costa Rica	3.4
Other countries	8.3
*Religious affiliation (%)*	
Catholic	70.7
Protestant	6.2
Other	4.0
None	19.1
*Believing person (%)*	
Not at all	15.8
Rather not	24.0
Yes, somehow	33.0
Yes, indeed	27.2
*Praying (%)*	
Not at all	17.8
Rarely	32.4
Occasionally	36.8
Regularly	12.9
*Meditation (%)*	
Not at all	22,6
Rarely	48.0
Occasionally	24.5
Regularly	4.9

*Due to multiple response options, numbers may exceed 100%

Catholics were predominating (71%), while 19% declared no religious affiliation. Sixty percent considered themselves as a believer (or somehow). Of these believers, 86% were Catholics and 13% had other religious affiliations, while among the nonbelieving persons, 68% were nominally Catholics, and 9% had other religious denominations; 50% of the sample were praying occasionally or even regularly, and 29% were meditating occasionally or regularly (Table 1).

### Exploratory Factor Analysis

The validation process of the Spanish language version of *Spiritual Care Competence Questionnaire* (SCCQ) follows the established procedures which are summarized by Beaton et al. (2000) and Koenig and Al Zaben (2021).

For exploratory factor analysis of the data pool, we relied on 744 complete SCCQ datasets. Internal consistency of the 26 items was primarily very good (Cronbach’s alpha = 0.923). Explorative principal component analysis revealed a Kaiser–Mayer–Olkin value of 0.91, which is a measure for the degree of common variance, indicating the item pool’s suitability for statistical investigation by means of principal component analysis. During this process, three items were eliminated: Two items loaded weak on factor 1, but they belonged to the factor *Knowledge about other religions* (items s28 and s43), and one item loaded weakly on factor 3 (item s8).

The 23 remaining items differentiated in six factors with eigenvalues of > 1.0. These accounted for 67% of variance (Table 2). Beside two items in Factor 1, which were the primary topic *Knowledge about other religions*, all other factors remained stable. Internal consistency of the six factors was good (Cronbach’s alpha ranging from 0.71 to 0.90). The three-item factor *Documentation competences* had the highest internal consistency, and the item factor *Self-awareness competences* the lowest (Table 2).

**Table 2:** Factorial structure of the 23-item SCCQ in its Spanish version

	Mean value [0–3]	Difficulty index (mean/3 = 0.59	Corrected item—scale correlation	Cronbach’s alpha if item deleted (alpha = .911)	Factor loading					
					1	2	3	4	5	6
**Factor 1: Empowerment competences** (Eigenvalue 34.7, Cronbach’s alpha = .816)										
25 In the case of therapeutic decisions, I pay attention to religious/spiritual attitudes and convictions of the individual patient	2.27 ± 0.88	0.76	.507	.908	.758					
39 I take care that the religious characteristics of patients from other religious communities are adequately considered	2.14 ± 0.88	0.71	.508	.908	.747					
26 I encourage my patients to reflect their spiritual beliefs and attitudes	2.44 ± 0.78	0.81	535	.907	.671					
38 I am well aware of the religious characteristics of patients from other religious communities	1.76 ± 0.92	0.59	.526	.907	.599					
24 I enable my patients to participate in religious activities/celebrations	1.41 ± 1.12	0.47	.448	.909	.509					
35 I pay attention to the appropriate framework for spiritual conversations	1.98 ± 0.94	0.66	.598	.906	.490					
43 I open verbally, but also nonverbally, a “space” in which the patient may bring spiritual concerns—but is not forced to do so	1.98 ± 0.95	0.66	.650	.905	.461					
**Factor 2: Conversation competences** (Eigenvalue 8.8, Cronbach’s alpha = .800)										
19 I am able to conduct an open discussion on existential issues	2.51 ± 0.76	0.84	.373	.910		.889				
20 I am able to conduct an open discussion on religious issues	2.48 ± 0.75	0.83	.378	.910		.860				
**Factor 3: Perception of spiritual needs** (Eigenvalue 1.8, Cronbach’s alpha = .808)		0.00								
1 I am confident I can perceive the spiritual needs of patients	2.31 ± 0.68	0.77	.467	.909			−.937			
2 I am confident I can perceive the spiritual needs of patients’ relatives	2.21 ± 0.73	0.74	.508	.908			−.903			
7 I am able to perceive existential/spiritual needs even if patients have little relation to religion	1.92 ± 0.92	0.64	.594	.906			−.484			
**Factor 4: Spiritual self-awareness competences** (Eigenvalue 1.4, Cronbach’s alpha = .706)										
48 I regularly take care of deepening my own spirituality	1.80 ± 1.03	0.60	.461	.909				.822		
30 My own spirituality shapes my dealings with others/sick people	1.96 ± 1.04	0.65	.364	.911				.740		
49 I regularly attend professional development sessions on spiritual topics	1.12 ± 1.08	0.37	.524	.907				.700		
**Factor 5: Team spirit** (Eigenvalue 1.2, Cronbach’s alpha = .868)										
13 In our institution there is a great openness to the topic of spirituality	1.63 ± 1.09	0.54	.517	.908					.875	
14 In the team, we exchange regularly about spirituality in patient support	1.35 ± 1.09	0.45	.688	.903					.821	
15 In the team, we regularly exchange about our own spirituality	1.25 ± 1.02	0.42	.593						.796	
12 In our team, we speak regularly about the spiritual needs of the patients	1.33 ± 1.09	0.44	.656	.904					.743	
17 In the team, we have rituals (for example, farewell and interruption rituals) to deal with problematic situations	1.03 ± 1.06	0.34	.424	.910					.635	
**Factor 6: Documentation competences** (Eigenvalue 1.0, Cronbach’s alpha = .902)										
4 I am familiar with instruments/questionnaires for structurally assessing spiritual needs	1.13 ± 1.07	0.38	.623	.905						−.905
3 I am familiar with instruments (i.e., topic list) for creating a short spiritual history	1.42 ± 1.08	0.47	.610	.905						-.887
5 I know how to document the spiritual history of my patients in a comprehensible way	1.22 ± 1.01	0.41	.635	.905						-.839
**Excluded Items**										
8 I can also talk with nonreligious patients about their existential/spiritual needs	1.95 ± 0.98	0.65								
28 I am able to tolerate the pain/suffering of patients and their relatives	2.34 ± 0.70	0.78								
42 I regularly approach patients to talk with them about their spiritual needs	1.43 ± 1.00	0.48								

Extraction method: Principal component analysis using Oblimin rotation with Kaiser normalization (converged in 9 iterations)

The six factors explain 67% of variance

The item difficulty index of the 23 items (1.77 [mean of included items] / 3 = 0.59) lies in the acceptable range (0.2 and 0.8). Three items showed ceiling effects (item s19*, I am able to conduct an open discussion on existential issues*; s20, *I am able to conduct an open discussion on religious issues*; s26*, I encourage my patients to reflect their spiritual beliefs and attitudes*), none had floor effects.

### Confirmatory Factor Analysis

As the structure of the Spanish version was quite similar compared to the German version, we performed a confirmatory factor analysis with the same data pool. After mathematical confirmation of the adequacy of the dataset to perform a factor analysis (p-value from Bartlett’s test p < 0.001 and KMO = 0.92), the confirmatory factor analysis (cfa) with the maximum likelihood estimator was run. The model fit values were CFI = 0.96, TLI = 0.95, RMSEA = 0.04 and SRMR = 0.04, which are considered acceptable. Factor F1 correlated moderately with all other factors (${r}_{F1,F2}=0.53$, ${r}_{F1,F3}=0.70$, ${r}_{F1,F4}=0.65$, ${r}_{F1,F5}=0.63$ and ${r}_{F1,F6}=0.57$) as also factors F3 and F6 (${r}_{F3,F6}=0.63$) and F5 with F6 (${r}_{F5,F6}=0.56$).

**Fig. 1:**
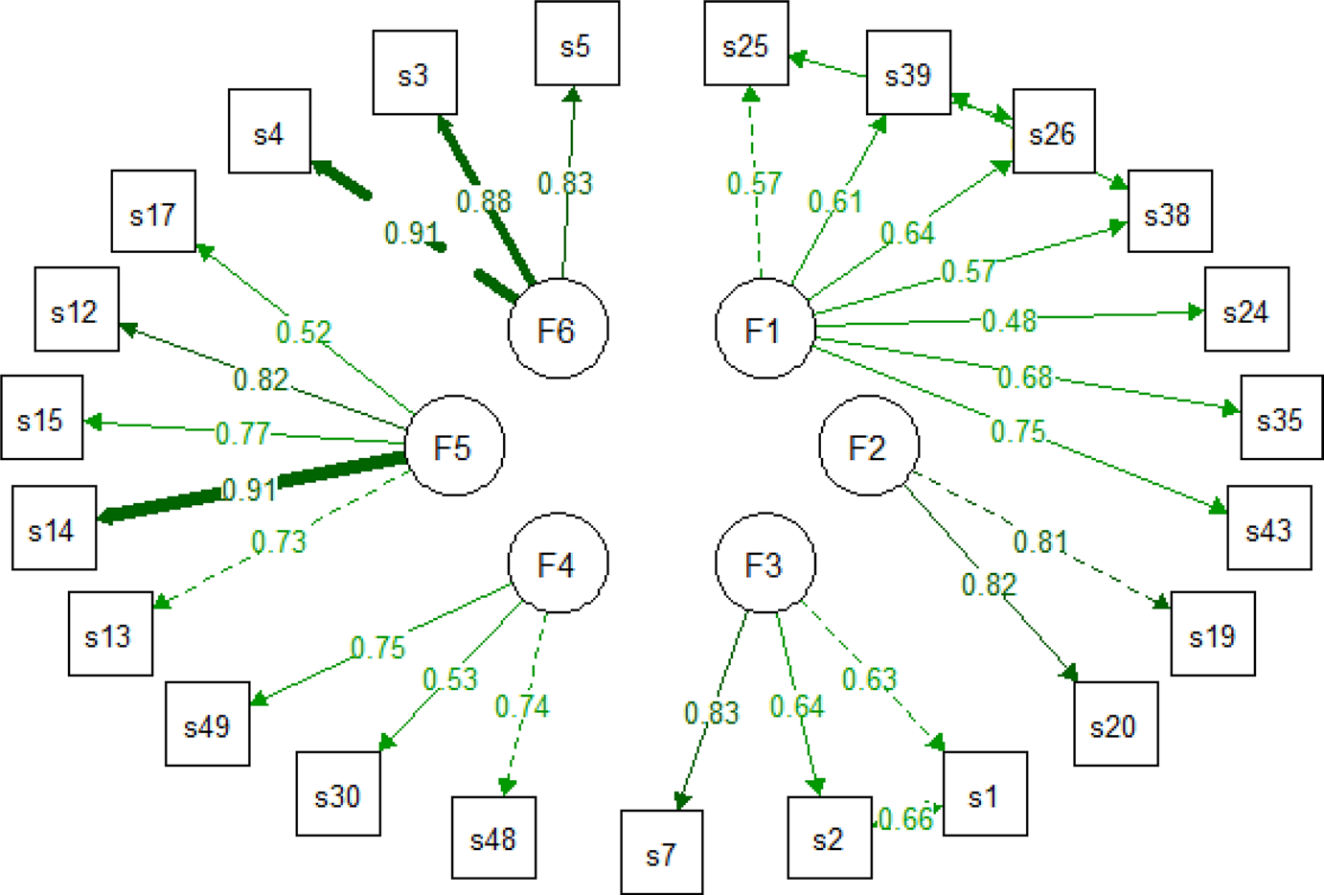
Confirmatory factor analysis for the data, factor loadings are displayed with one directional arrows from factors to variables and correlations are displayed with two bidirectional arrows between variables

The items with significant correlation for the model were s1 with s2 (${r}_{s1,s2}=0.66$), s38 with s39 (${r}_{s38,s39}=0.31$) and s25 with s26 (${r}_{s25,s26}=0.32$). According to the factor loadings, the strongest associations were found in factor F5 with variable s14 (0.91) and in factor F6 with variables s3 (0.88) and s4 (0.91), see Fig. 1.

### Distribution of SCCQ Scores in the Sample

*Conversation competences* and *Perception of spiritual needs competences* scored highest (2.5 ± 0.7), and *Documentation competences* (1.3 ± 1) and *Team spirit* the lowest (1.3 ± 0.9). *Conversation competences* scored statistically significantly higher in men compared to women (2.5 ± 0.7 vs. 2.6 ± 0.6), while in all other SCCQ scores no significant differences were observed (Table 3).

**Table 3:** Distribution of SCCQ scores in the sample

		Empowerment competences	Conversation competences	Perception of spiritual needs	Spiritual self-awareness competences	Team spirit	Documentation competences	Hindrances
All participants	Mean	1.99	2.49	2.15	1.62	1.32	1.26	1.16
	SD	0.65	0.69	0.67	0.84	0.86	0.96	0.76
*Gender*								
Women	Mean	2.01	2.45	2.13	1.64	1.29	1.23	1.18
	SD	0.64	0.70	0.67	0.82	0.87	0.96	0.76
Men	Mean	1.95	2.60	2.20	1.57	1.38	1.32	1.09
	SD	0.67	0.63	0.67	0.87	0.85	0.95	0.75
*F* value		1.48	**6.88**	1.98	1.14	1.69	1.27	2.09
*p* value		n.s	**0.009**	n.s	n.s	n.s	n.s	n.s
*Religious believer*								
Nonbelieving person	Mean	1.86	2.46	2.02	1.27	1.14	1.13	1.24
	SD	0.62	0.72	0.69	0.80	0.83	0.95	0.75
Believing person	Mean	2.10	2.51	2.21	1.89	1.47	1.35	1.12
	SD	0.62	0.68	0.64	0.75	0.87	0.96	0.77
*F* value		**24.52**	0.84	**13.84**	**109.59**	**26.11**	**8.41**	4.32
*p* value		** < 0.0001**	n.s	** < 0.0001**	** < 0.0001**	** < 0.0001**	**0.004**	0.038
*Department*								
Palliative care	Mean	2.30	2.64	2.30	1.78	1.73	1.67	0.94
	SD	0.48	0.54	0.58	0.81	0.81	0.88	0.68
All other	Mean	1.81	2.41	2.06	1.53	1.08	1.02	1.28
	SD	0.66	0.75	0.70	0.84	0.80	0.93	0.77
*F* value		**121.03**	**21.02**	**25.19**	**15.99**	**121.96**	**93.78**	**38.63**
*p* value		** < 0.0001**	** < 0.0001**	** < 0.0001**	** < 0.0001**	** < 0.0001**	** < 0.0001**	** < 0.0001**

Significant differences *p* < 0.0001 are highlighted (bold); n.s. = not significant

Except for *Conversation competences*, all other competences were significantly higher in persons who state as believers compared to nonbelievers. Participants working in palliative care reported significantly higher SCCQ scores in all competences compared to participants working in other fields (Table 3).

### Correlations between SCCQ Factors and Sociodemographic Variables

All other competences were moderately to strongly interrelated with the exception of *Conversation competences*, which was better correlated with *Perception of spiritual needs competences,* but weakly correlated with other dimensions and negligible with *Documentation competences* (Table 4).

**Table 4:** Correlations between SCCQ factors and other variables

	Empowerment competences	Conversation competences	Perception of spiritual needs	Spiritual self-awareness competences	Team spirit	Documentation competences	Hindrances
Empowerment competences	1.000						
Conversation competences	.391^*^	1.000					
Perception of spiritual needs	.490^*^	.315^*^	1.000				
Spiritual self-awareness competences	.479^*^	.260^*^	.344^*^	1.000			
Team spirit	.534^*^	.213^*^	.369^*^	.354^*^	1.000		
Documentation competences	.471^*^	.134^*^	.521^*^	.389^*^	.492^*^	1.000	
Hindrances	−.469^*^	−.284^*^	−.404^*^	−.350^*^	−.360^*^	−.321^*^	1.000
Age	.175^*^	.023	.259^*^	.251^*^	.198^*^	.179^*^	−.124^*^
Years in the job	.104^*^	.010	.229^*^	.169^*^	.143^*^	.129^*^	−.091
Duration of work per week	−.050	−.023	−.082	−.054	−.100^*^	−.077	.127^*^
Job satisfaction	.241^*^	.136^*^	.241^*^	.155^*^	.300^*^	.136^*^	−.158^*^
Frequency of praying	.255^*^	.094	.135^*^	.374^*^	.202^*^	.138^*^	−.152^*^
Frequency of meditation	.159^*^	.081	.241^*^	.325^*^	.143^*^	.152^*^	−.177^*^

^*^p < 0.001 (Spearman rho); Moderate to strong correlation are highlighted (bold)

Frequency of praying or meditation was positive moderately related to *Spiritual self-care competences*. Praying was weakly related to *Empowerment competences* and meditation marginally only, while meditation was weakly related to *Perception of spiritual needs competences* (Table 4).

Job satisfaction showed a moderate correlation with *Team spirit* and a weak correlation with *Empowerment competences* and *Perception of spiritual needs.* Age and work experience were only weakly related to *Perception of spiritual needs competences.* Age also showed a weak correlation with *Spiritual self-awareness competences.* Weekly working time showed no significant correlations with most of the competencies (Table 4).

## Discussion

This large sample of health care professionals from Spanish speaking countries reflects the great interest in the subject of spiritual care competence. Although we had four target countries, being an open survey and circulating in networks, it reached professionals from other countries, and we decided to include them in the analysis. The scale translation covers in great part the international recommendations for religious and spiritual measures (Koenig & Al Zaben, 2021).

### Validation of the SCCQ

We were able to verify the same six-factor structure of the original German version of SCCQ (*Empowerment competences*, *Conversation competences*, *Perception of spiritual needs*, *Spiritual self-awareness competences*, *Team spirit* and *Documentation competences*). After eliminating 3 items, the main differences compared to the original version was that factor *Empowerment competences* that included two items of the factor *Knowledge about other religions*, and one item from the *Spiritual Self-awareness competence* scale. All other items loaded on the same factors as in the primary German language version, and also the internal consistency of all factors was similar compared to the original version (Frick et al., 2019), and the structure was confirmed by CFA, which indicates an appropriate adaptation process.

### Spiritual Competences

*Conversation competences* was the highest scored factor, similar to that obtained in the German sample. Both groups scored the competences in the same sequence. The Spanish speaking participants in this study reported higher scores in all competences as the German sample (Frick et al., 2019), especially in *Documentation competences* and *Spiritual self-awareness competences*.

Special training should be oriented especially to *Documentation competences* which obtained the lowest score (also in the German sample). *Team spirit* also ranked low; however, it might be necessary to take in account the working conditions. Educational activities were shown to be useful in enhancing confidence in delivering spiritual care and providing a context to examine their own spirituality (Jones et al., 2021; Kalish, 2012; Paal et al., 2015).

Barriers/hindrances were perceived less often in palliative care units compared to other departments, which may be due to training differences. Hindrances were perceived in trend marginally stronger by nonbelievers which seems to be less willing to address these issues. In fact, these hindrances correlated inversely with all spiritual care competences as one may theoretically assume, too. Perceiving lot of hindrances might be an unconscious strategy to not address spiritual issues in routine work, particularly by health care professionals who may feel unpleasant with this topic.

### Associated Factors

Participants who considered themselves as “believers” (regardless of their practices) reported significantly higher scores in all competences (with the exception of *Conversation competences*), with higher scores compared to the nonbelievers. Likewise, the amount of spiritual praxis (praying/meditation) was also positively and moderately correlated with *Spiritual self-awareness competences.* In the German sample, no associations were found among these variables. Spiritual practices such as praying or meditating were moderately related to *Spiritual self-awareness competences*, as in the German sample.

Palliative care is a field in development in Latin America and the amount of professionals in this area is reduced (Pastrana & De Lima, 2021); however, they constituted over one third of the sample. Participants with a palliative care background scored significantly higher on all spiritual care competences than professionals from other disciplines. Probably, because in the palliative care tradition the holistic approach is taken very seriously, and spirituality is considered an intrinsic and essential component (Gijsberts et al., 2019; Kunsmann-Leutiger et al., 2021).

General practitioners were the second more numerous group of participants. According to the literature, they are responsible for assessing the patients' spiritual needs and responding to it, but frequently struggle with the spiritual terminology and are both uncomfortable and afraid that patients will refuse to participate in the dialogue (Kunsmann-Leutiger et al., 2021; Vermandere et al., 2011).

### Limitations

Although the translation process was rigorous, including experts in selected countries and searching for a clear formulation while conserving the original meaning, there may exist differences in the interpretation or understanding in the statements between countries in the same subregion or even within countries.

Other limitations of this study are those inherent to any cross-sectional designs. Also, the use of a convenience sample and the voluntary participation of respondents, a selection effect for professionals interested in spirituality is possible. In fact, the sample does not represent the distribution of health care professionals in Latin America; for example, the predominance of physicians as well as participants working in palliative care can be explained due the network of the authors. For future studies, we intend to enroll a weighted sample of professionals. Despite these limitations, the study allowed the validation process and provided a glimpse into the spiritual care competences in the region.

Finally, the instrument only assessed self-reported competences. Although the act of self-assessment is an intrinsically difficult task, spiritual care competencies might be overestimated due to a variety of psychological mechanisms (Dunning et al., 2004). Despite that the perceived competence might not reflect the actual performance, the different items of the instrument may present a balanced picture.

## Conclusions

In conclusion, this Spanish language version was very similar to the original German language instrument. Since Spiritual Care is influenced by attitudes and personal convictions, as well individual and social skills, the SCCQ primarily portrays spiritual care competencies, to detect training needs and to plan for specific training support measures for the health professions. The SCCQ can be used for comparisons as well as monitoring/follow-up after implementation of improvement strategies, as educational activities.

Based on the results, we recommend the development of guidelines and design of a basic training in spiritual care for health care provides based on the identified competences to address both, spiritual distress and spiritual needs (Büssing, 2021). So that interprofessional spiritual care would be fully integrated and meeting the patients’ needs.

Further research about effectivity of educational training in spiritual care as well as about the perspective of patients and families regarding the meet spiritual need and satisfaction would be necessary.

## Data Availability

According to the data protection regulations, the dataset cannot be made publicly available. Data are, however, available from the authors upon reasonable request (www.spiritual-competence.net).
